# Variability in sea ice cover and climate elicit sex specific responses in an Antarctic predator

**DOI:** 10.1038/srep43236

**Published:** 2017-02-24

**Authors:** Sara Labrousse, Jean-Baptiste Sallée, Alexander D. Fraser, Rob A. Massom, Phillip Reid, William Hobbs, Christophe Guinet, Robert Harcourt, Clive McMahon, Matthieu Authier, Frédéric Bailleul, Mark A. Hindell, Jean-Benoit Charrassin

**Affiliations:** 1 Sorbonne Universités, UPMC Univ., Paris 06, UMR 7159 CNRS-IRD-MNHN, LOCEAN-IPSL, 75005 Paris, France; 2 Marine Predator Unit, Institute for Marine and Antarctic Studies, University of Tasmania, Private Bag 129, Hobart, Tasmania 7001, Australia; 3 British Antarctic Survey, High Cross, Cambridge, CB3 0ET, UK; 4 Institute of Low Temperature Science, Hokkaido University, N19 W8, Kita-ku, Sapporo 060-0819, Japan; 5 Antarctic Climate & Ecosystems Cooperative Research Centre, University of Tasmania, Private Bag 80, Hobart, Tasmania 7001, Australia; 6 Australian Antarctic Division, Channel Highway, Kingston, Tasmania 7050, Australia; 7 Australian Bureau of Meteorology, Centre for Australian Weather and Climate Research, Hobart, Tasmania 7001, Australia; 8 Centre of Excellence for Climate System Science, Australian Research Council, Sydney, New South Wales 2052, Australia; 9 Centre d’Etudes Biologiques de Chizé (CEBC), UMR 7372 Université de la Rochelle-CNRS, 79360 Villiers en Bois, France; 10 Department of Biological Sciences, Macquarie University, Sydney, New South Wales 2109, Australia; 11 Sydney Institute of Marine Science, 19 Chowder Bay Road, Mosman, New South Wales 2088, Australia; 12 Observatoire PELAGIS, UMS 3462 CNRS-ULR, 17000 La Rochelle, France; 13 South Australian Research & Development Institute (SARDI), 2 Hamra Avenue, West Beach, South Australia 5024, Australia

## Abstract

Contrasting regional changes in Southern Ocean sea ice have occurred over the last 30 years with distinct regional effects on ecosystem structure and function. Quantifying how Antarctic predators respond to such changes provides the context for predicting how climate variability/change will affect these assemblages into the future. Over an 11-year time-series, we examine how inter-annual variability in sea ice concentration and advance affect the foraging behaviour of a top Antarctic predator, the southern elephant seal. Females foraged longer in pack ice in years with greatest sea ice concentration and earliest sea ice advance, while males foraged longer in polynyas in years of lowest sea ice concentration. There was a positive relationship between near-surface meridional wind anomalies and female foraging effort, but not for males. This study reveals the complexities of foraging responses to climate forcing by a poleward migratory predator through varying sea ice property and dynamic anomalies.

Over the last 30 years, Earth’s polar regions have experienced significant changes in their sea ice coverage, with predictions of accelerated future change in the coming century^1^. The Southern Ocean has already undergone large regionally-contrasting trends in sea ice coverage over the last 30 years. This is characterized by gain in the Ross Sea and loss in the neighbouring Amundsen/Bellingshausen Seas sectors^2^,^3^, with patterns of change/variability across the extensive East Antarctic sector being more spatially complex^4^. Sea ice-covered regions represent a unique and highly productive habitat and in the face of these large changes, ice coupled ecosystems experience re-organization associated with rapid change of their habitat^5^. Change in ecosystem structure and function may translate into modification of top predator population dynamics, because top predators integrate the spatio-temporal variations in underlying trophic levels^6^. Long-term studies have recently started to quantify the relationships between top predator population dynamics and inter-annual variability in sea ice concentration and extent (e.g. refs 7, 8, 9, 10, 11, 12, 13, 14, 15). Observed responses are not uniform among populations and species around Antarctica^5^, yet much remains to be understood about how individual animals use their environment, and how both environmental and associated food-web changes, effect their foraging performance at-sea, and ultimately their population dynamics. In this study, we can address this as we have collected a unique 11-year time-series of coupled sea ice and seal behavioural observations. We present novel results on the foraging behaviour of southern elephant seals (*Mirounga leonina*; SESs) according to regional variability in sea ice and wind patterns across East Antarctica.

SESs are deep-diving, wide-ranging predators^16^, and major consumers of marine resources of the Southern Ocean^17^,^18^, they depend upon an extensive set of trophic levels within the marine food web. They utilize different marine habitats depending on their sex^19^,^20^ and their breeding colony locations^21^. For these reasons, SESs are unique model species to investigate physical changes over wide spatio-temporal ranges and they provide an unprecedented opportunity to integrate behaviour and physical structure to quantify how animals respond to variation in their environment. As a non sea ice-obligate species, SESs are often under-represented in ecological sea ice studies, yet they strongly interact with sea ice during their Antarctic foraging trips^20^,^21^,^22^,^23^,^24^,^25^. The under-ice environment supports a rich winter food resource, providing both a substrate for the growth of ice algae and a refuge for herbivorous zooplankton such as juvenile krill and other crustaceans^26^,^27^,^28^,^29^, which in turn attracts higher trophic levels such as pelagic fish and their predators^30^,^31^,^32^,^33^,^34^. Inter-annual changes in both regional sea ice concentration and the timing of sea ice advance may therefore affect the availability of resources within the sea ice zone^5^, but no studies have assessed the foraging response of diving predators to such change/variability.

Around much of Antarctica, variability in sea ice concentration and the timing of annual sea ice advance (and retreat) is linked with variability in wind patterns as they affect both sea ice dynamic and thermodynamic processes. While cold southerly winds tend to drive enhanced equatorward ice advance and (depending on the season) increase the ice concentration, warmer northerly winds can compact the sea ice^35^,^36^,^37^,^38^,^39^,^40^. In East Antarctica, recent analyses have shown that changes in sea ice contain a strong wind-driven thermodynamic component^39^. Coupled sea ice model experiments depict a strong, non-annular response of wind-induced sea ice drift in East Antarctica, with strong westerlies leading to increased sea ice concentration in the western part of this region while further east, a strong northerly wind results in decreased sea ice concentration^40^. Aspects of these winds and sea ice changes are associated with trends in large-scale climate modes of variability such as the Southern Annular Mode (SAM)^35^,^40^,^41^,^42^, which itself is forced by the Southern Hemisphere ozone hole and increased greenhouse gases^38^,^43^.

In this study, we examine an 11-year time-series (2004–2014) of SES movements and diving behaviour to quantify how wind variability and the associated sea ice variability, both forced by large-scale climate variability, affect top predator foraging activity through abiotic and biotic mechanisms. Previous work on this dataset has shown that adult females prefer to forage in high sea ice concentration regions, close to the sea ice edge in the pack ice, while juvenile males remain deep within the sea ice to forage mainly over the Antarctic shelf or within the Antarctic Slope Front and in low sea ice concentration regions (presumably polynya areas)^20^,^21^,^23^,^25^. In the present paper, we show for the first time how this sex-dependent habitat utilization is affected by inter-annual variability in sea ice in East Antarctica. In particular, we highlight the role of near-surface meridional winds, incorporating large-scale climatic variability, in impacting predators through their effects on regional sea ice changes. The effect of the timing of sea ice advance on seal foraging performance brings new insights to the underlying seasonal trophic mechanisms by which sea ice is critical to Antarctic ecosystems right through to predators.

## Results

### Seal foraging strategy and sea ice habitat

**Figure 1:**
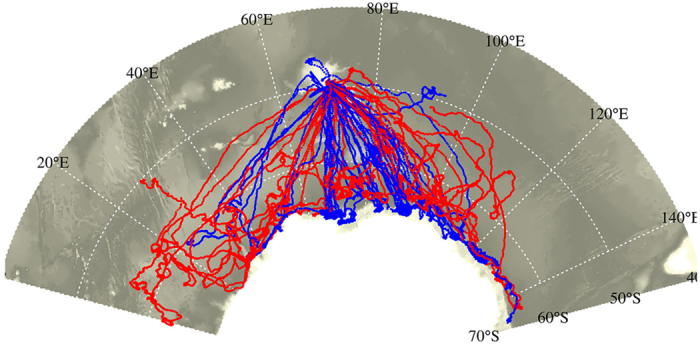
Tracks of the 43 southern elephant seals equipped with CTD-SRDLs from 2004 to 2014. Their movements and diving behaviour were collected during their post-moult foraging trip from the breeding colony in Kerguelen Islands to the Antarctic sea ice zone. Red and blue colours represent the 21 females and 22 males, respectively. The map was made using R software, version 3.2.4 revised (R Core Team (2016). R: A language and environment for statistical computing. R Foundation for Statistical Computing, Vienna, Austria. URL https://www.R-project.org/). The bathymetry represented in grey shading is from The GEBCO_08 Grid, a global 30 arc-second grid largely generated by combining quality-controlled ship depth soundings with interpolation between sounding points guided by satellite-derived gravity data. URL http://www.gebco.net/data_and_products/gridded_bathymetry_data/.

Winter post-moult foraging trips of 43 SESs (21 females and 22 males for a total of 273,542 dives) from Kerguelen Islands to the seasonal Antarctic sea ice zone were monitored using satellite-relayed position and diving data from 2004 to 2014 (Fig. 1; Supplementary information, Table S1). Previously we identified two foraging strategies among post-moult Kerguelen SESs: open ocean foragers that predominantly use the Kerguelen shelf or frontal regions of the Antarctic Circumpolar Current (ACC), and high Antarctic specialists that forage mainly in the sea ice covered seas in close proximity to the Antarctic continent^19^,^21^. In this study we focus on the latter group of seals.

The tracked seals spanned a large region longitudinally ranging from 0 to 150°E (Fig. 1), which can be divided into three sectors with distinct sea ice cover characteristics^4^: (i) from 0 to 50°E, the winter sea ice cover has a large latitudinal range relatively early in the season (before March/April), largely driven by net sea ice production within sea ice and at the sea ice edge^44^ and supplemented by an eastward transport of sea ice from strong westerlies (during positive SAM events)^40^ and within the eastern Weddell Gyre^44^; (ii) from 50 to 90°E, the sea ice cover also extends far to the north, with a number of coastal polynyas producing large amounts of sea ice^45^ which is transported offshore by a net northward winds and the Prydz Bay Gyre, both within the climatological low-pressure Amery Bay region^40^; and (iii) from 90 to 150°E, a narrower zone of sea ice which is mostly fed by production in coastal polynyas and leads and supplemented by advection (input) from the east^4^,^44^. Wind convergence (i.e. stronger northerly wind component during positive SAM events) in the eastern part can locally limit the sea ice extent resulting in compacting ice at the coast^37^,^40^.

**Figure 2:**
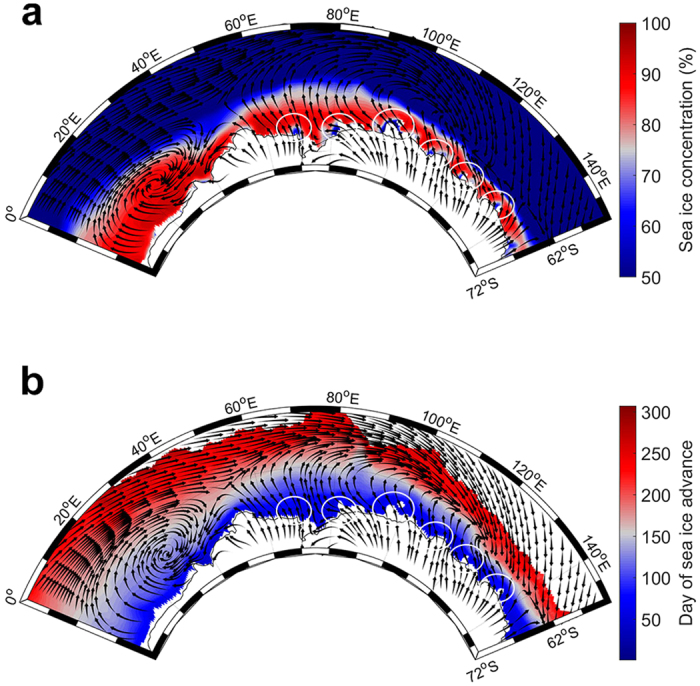
Climatological patterns of sea ice and near-surface winds from 2004 to 2014. **(a)** Mean sea ice concentration (expressed as percentage) and monthly-averaged ERA-Interim 10 m winds zonal and meridional component are shown for the winter season (June-August). **(b)** Mean day of sea ice advance and monthly-averaged ERA-Interim 10 m winds zonal and meridional component from March to June. Ellipses represent coastal polynya sites, from left to right: Cape Darnley/Mackenzie, Barrier, Shackleton, Vincennes Bay, Dalton, and Dibble. For illustration purposes, autumn-averaged sea ice concentration (March-May) is not represented. Maps were made using MATLAB software (version 8.5.0.197613 (R2015a), URL http://fr.mathworks.com/).

The Fig. 2 illustrates the averaged sea ice cover, advance and near-surface wind patterns from 2004 to 2014 during the winter season for the study region. In each of sectors described above, the mean wind field is consistent with the mean sea ice cover and day of advance. We also observed in Fig. 2 the processes described above: concentrated sea ice and advance extending far north due to the eastward transport of sea ice in the first sector and to net northward winds in the second sector; a narrow sea ice zone due to wind convergence in the third sector; and generally over the whole study region stronger mean northward wind driving earlier sea ice advance in the pack ice region.

### Seal foraging activity response to inter-annual sea ice cover anomaly

The mean sea ice concentration and day of sea ice advance exhibit large inter-annual variability across East Antarctica^4^. To investigate how seals respond to this, we divided the individual dives into two groups; those foraging during strongly positive and negative sea ice concentration anomalies, for both males and females (see Methods section). The combined number of seal observations in the two groups of sea ice concentration anomalies comprised about 9% of the total female dives (n = 12,694 dives, 17 females) and 12% of the total male dives (n = 15,996 dives, 21 males). Similarly, we defined two further groups of dives corresponding to earlier or later sea ice advance (as opposed to concentration), for both males and females (see Methods section). The combined number of seal observations in the two groups of sea ice advance anomaly was 58,906 dives, comprising about 25% of the total female dives (n = 35,038, 14 females) and about 18% of the total male dives (n = 23,868, 19 males).

**Figure 3:**
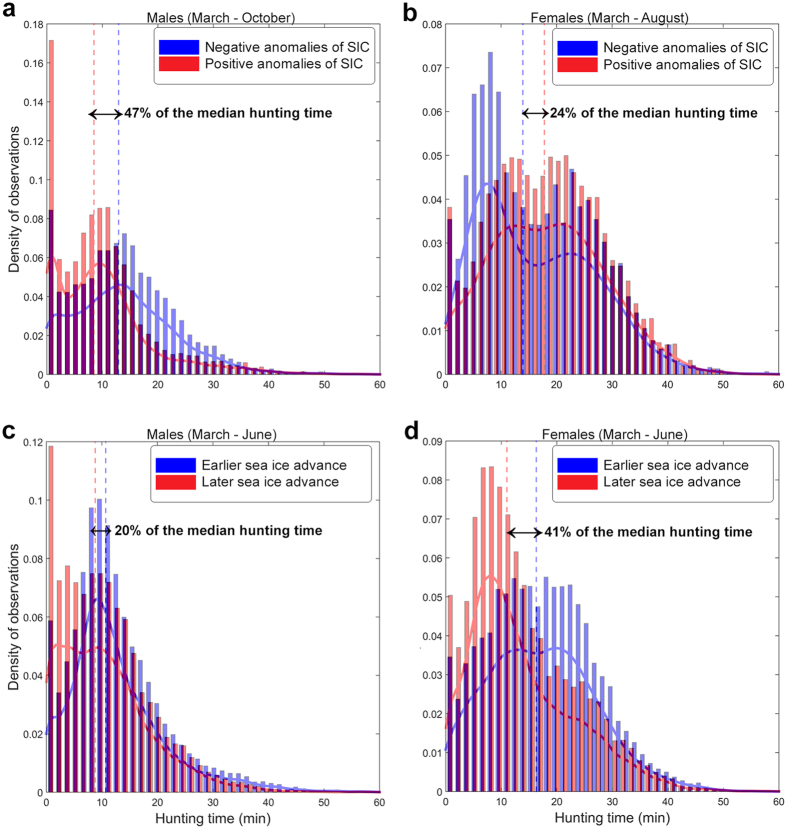
Influence of sea ice changes on male and female foraging activity from 2004 to 2014. Normalized histograms of the sum of observations in each bin of hunting time (*i.e.* a proxy of seal foraging activity expressed in minutes) are represented for negative or positive sea ice concentration (SIC) anomalies (see Methods) for **(a)** males and **(b)** females. The same histograms are presented for earlier and later advance of sea ice for **(c)** males and **(d)** females. For each group of anomalies, the probability density function was superimposed and the dashed lines represent the median hunting time for each group of anomalies for males and females. Please note that hunting times equal to 0 were removed for illustration purposes.

For males, hunting times were on average 4.6 min/dive longer (or 47% of the median hunting time, n = 21) when sea ice concentration was lower (negative) than when positive sea ice concentration anomalies were observed (Table 1; Fig. 3a). The maximum difference in hunting times between two generated random groups of sea ice concentration anomalies from the bootstrap analysis was 0.6 min/dive, i.e. ~8 times lower than the test condition (*p-value* ≈ 0), confirming the significance of negative sea ice concentration anomalies in influencing male hunting times. Hunting times were 1.9 min/dive longer (or 20% of the median hunting time, n = 19; Table 1; Fig. 3c) in years with earlier sea ice advance. Using a bootstrap analysis, the maximum difference in hunting times between two generated random groups of earlier and later sea ice advance was 0.5 min/dive, i.e. ~4 times lower than the test condition. Thus, the difference between earlier and later sea ice advance was significant (*p-value* ≈ 0) but the impact of earlier sea ice advance for males was relatively low.

In contrast to males, positive sea ice concentration anomalies were associated with longer hunting times for females (i.e. 3.9 min/dive longer or 24% of the median female hunting time; Table 1; Fig. 3b). The bootstrap analysis showed a maximum difference in hunting times of 1.3 min/dive, *i.e.* ~3 times lower than the test condition. While the difference between sea ice concentration anomalies was significant (*p-value* ≈ 0), the impact of sea ice concentration anomalies on female hunting times was less important than for males. However, the effect of earlier sea ice advance on female hunting times was more marked, with their foraging time increasing by *~*5.3 min/dive, i.e. 41% of the median hunting time (Table 1; Fig. 3d). Bootstrap analysis confirmed this result for females: the maximum hunting time differences in median for randomly chosen groups of earlier and later advance was 0.6 min/dive, i.e. 9 times lower than the test condition, confirming the significance (*p-value* ≈ 0).

The effect of individual variability in the different analyses is presented in the supplementary information, figures S1 and S2.

### Inter-annual sea ice cover anomaly response to anomalous winds

**Figure 4:**
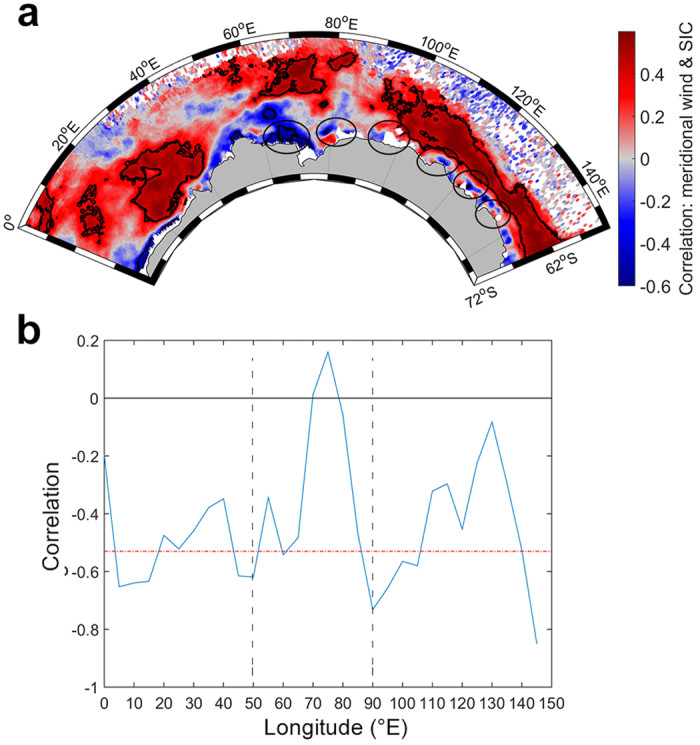
Relationship between 10 m wind meridional component and sea ice patterns from 2004 to 2014. Per-pixel Spearman correlation coefficient between monthly ERA-Interim 10 m wind meridional component anomaly and monthly sea ice concentration (SIC) anomalies from 2004 to 2014 is represented for the winter season (June-August), with contours denoting statistical significance at the 95% level. Ellipses represent coastal polynya sites, from left to right: Cape Darnley/Mackenzie, Barrier, Shackleton, Vincennes Bay, Dalton, and Dibble **(a)**. For each 5 degree longitude bin, monthly ERA-Interim 10 m meridional winds anomalies were averaged within the minimum and maximum latitude band of average day of advance from 2004 to 2014. The correlation values from Spearman correlation between sea ice advance anomalies and averaged 10 m wind anomalies meridional component for each 5° bin of longitude are represented by the blue line; the significance of the negative correlation is represented by the dotted red line **(b)**. For panel **(b)**, the two dotted black lines delineate regions of interest discussed in the text. For illustration purposes, autumn correlation for sea ice concentration (March-May) is not represented. Map in panel (**a**) was made using MATLAB software (version 8.5.0.197613 (R2015a), URL http://fr.mathworks.com/).

Both the local anomalies of sea ice concentration and advance effected seal foraging activity, with the ice anomalies being (at least partly) controlled by local near-surface winds^35^,^36^,^38^,^39^,^40^. Indeed, the correlation between local sea ice concentration and local winds anomalies (which are defined as deviation from mean seasonal cycle) over the time period examined (2004–2014) shows clearly differing wind-sea ice relationships across the different sectors of our study region (Fig. 4a). In the westernmost (0–50°E) and easternmost (90–150°E) sectors, significant positive correlations were found between near-surface northward wind and sea ice concentration anomalies in the ice-covered region (Fig. 4a). By contrast, in the intervening sector extending from 50°E and 90°E, there were significant negative correlations between sea ice concentration and near-surface northward wind anomalies. The largest negative correlation in this sector was found around the Mawson Coast/western Prydz Bay (60–75°E, Fig. 4a). This negative correlation suggests strong offshore transport of sea ice newly formed within the coastal polynyas. A similar and consistent impact of northward winds was also found for the day of sea ice advance (Fig. 4b). Negative correlations were found in the westernmost (0–50°E) and easternmost (90–150°E) sectors, with an increase in the northward component of the near-surface wind being associated with earlier sea ice advance. This contrasts with observations in the 60–75°E sector, where the larger the northward component of near-surface wind, the later the sea ice advance. This latter was presumably because of efficient zonal export of the early-formed sea ice, preventing sea ice from accumulating locally leading to later sea ice advance. These relationships were also found in all coastal polynyas (Fig. 4a). However, the presence of thin ice early in the season or multi-year sea ice in polynyas may lead to artefacts in the calculation of sea ice concentration and advance and consequently misinterpretation of the observed correlation.

### Indirect influence of local wind anomalies onto seal foraging activity

**Figure 5:**
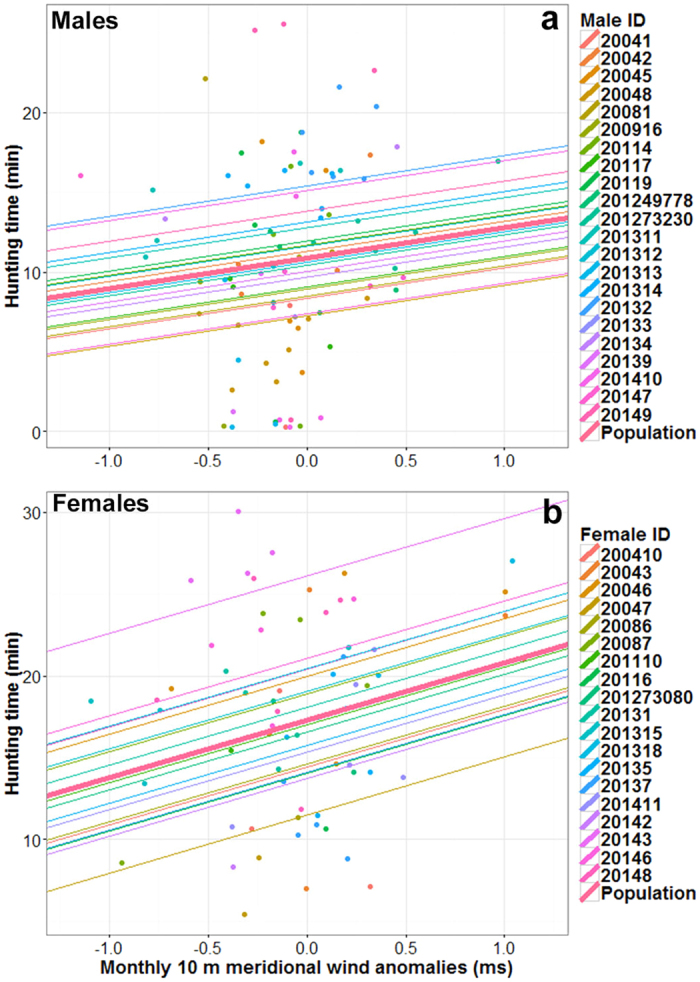
Relationships between foraging activity and meridional near-surface wind anomalies for (**a**) males and (**b**) females. Linear mixed effect models (LMMs) were used to quantify the links between foraging activity of males and females and monthly ERA-Interim 10 m meridional wind anomalies within the sea ice zone, from March to August for females and March to October for males. For each graph, the thick lines represent the predictive values from the population and the different thin lines represent the predictive values for each individual.

Our observations suggest that seal foraging activity is influenced by inter-annual sea ice anomalies, which are themselves a product of wind anomalies. Therefore we now investigate whether there is an indirect influence of wind on seal foraging activity. A linear mixed effects model (see Methods section) was used to investigate the relationship between wind anomalies and seal foraging activity. We found that there was a positive relationship (*t-value = 3.5, p-value = 0.001*) between the near-surface meridional wind anomalies and female hunting times, but not for male hunting times (*t-value = 1.1, p-value = 0.3*; Fig. 5a,b). Relationships were consistent among individuals. Linking hunting times at the dive scale (monthly averaged per individual) with monthly wind anomalies (monthly averaged per individual) at a coarse spatial resolution of approximately 80 km is appropriate as wind-driven sea ice changes occur at larger spatio-temporal scales than the dive scale. However, this also means that the present analysis presumably captures the global influence of wind on seal foraging activity through sea ice changes but not local changes in hunting times, and this may explain the relatively weak relationship (marginal R-squared of 11%).

## Discussion

In the present study, we assumed that increased hunting time generally indicates increased foraging success. This may be questionable given that increased hunting time may also be associated with the difficulty of finding resources. Hunting time encompasses foraging effort both at the bottom and during the transit phases of the dive and is well correlated with bottom time. Recent work from ref. 46 on high resolution dive data of SESs investigated the link between bottom duration and prey encounter events (PEE) derived from accelerometers. They found that for 90% of dives (successful dives, with at least one PEE), bottom time increased with the number of PEE at depth greater than 250 m; and beyond 550 m dive depth, bottom time starts decreasing with increasing dive depth regardless whether or not the dive was successful and unsuccessful (presence or absence of PEE). Therefore, for 90% of dives, the bottom time and in turn the hunting times are good indicators of foraging success (above 550 m dive depth). The validity of hunting time is thus dependent on diving depth, and below 550 m it may be biased as shorter bottom times (reflecting the physiological dive limits) may be associated with good foraging success. We found that the average dive depth of seals foraging within the sea ice region was around 400 m, so this bias may only concern deep dives within canyons along the Antarctic shelf or along the shelf break. Moreover, dive segments with hunting time were associated with a large proportion of PEE (68% of all PEE inferred from acceleration data) and with four times more PEE than other segments^47^. We are thus confident that this index is reliable for evaluating foraging activity of SESs within the sea ice region.

**Figure 6:**
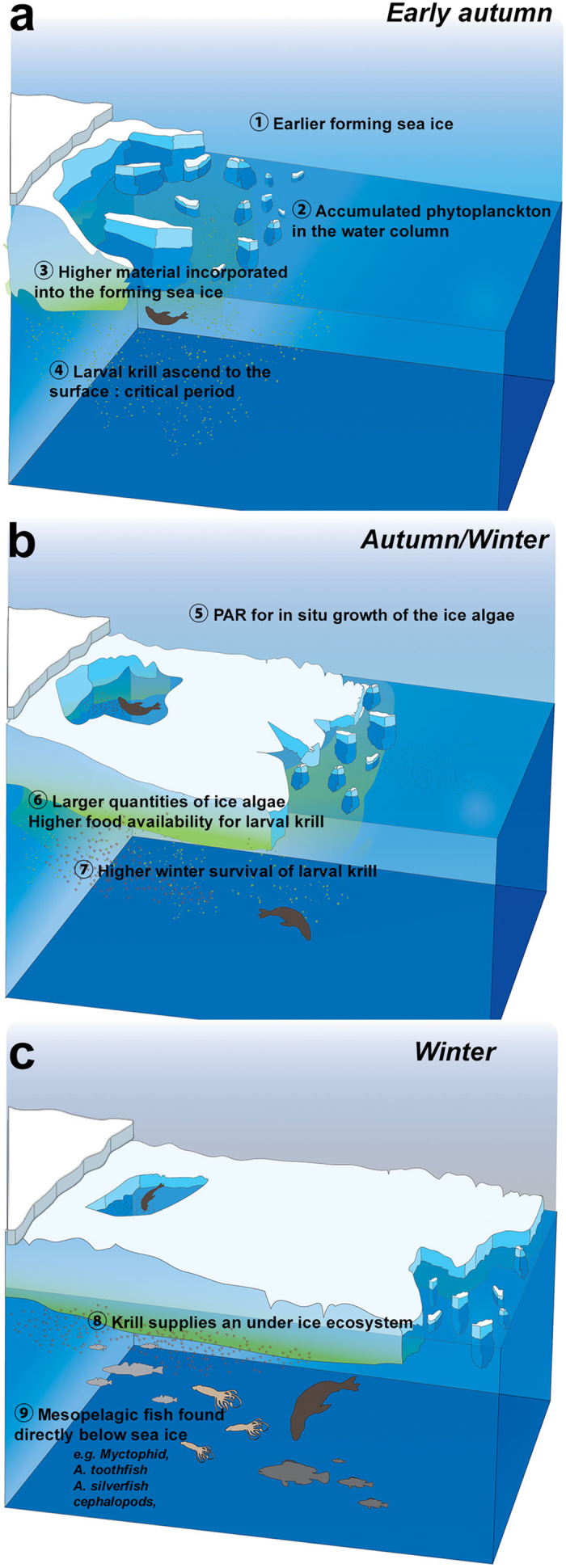
Schematic illustration of mechanisms underlying relationships between earlier sea ice advance and increased seal foraging activity. Conceptual model developed by refs 48, 50. A critical period is when sea ice advances in autumn at a time and location where larval krill ascend to surface waters, requiring food and refuge. The earlier the sea ice formation, the greater the amount of phytoplankton incorporated from the water column into the forming ice (**a**). The greater the amount if photosynthetically available radiation (PAR) for growth of the ice algae, the higher the food availability for krill, leading to higher survival rates in juvenile krill (**b**). In turn, krill supplies an under-ice ecosystem that favours SESs in winter. For example some mesopelagic organisms usually inhabiting deep water are found directly below sea ice in the pack ice areas (**c**) refs 32, 68, 69, 70. The illustration was made by Indi Hodgson-Johnston from Adobe Stock.

Favourable conditions for female foraging activity (i.e. longer hunting times) were observed for years of increased sea ice concentrations and earlier sea ice advance. We hypothesize that the early development and advance of sea ice in autumn would enhance primary production within the ice^5^,^48^ thereby providing increased resources for predators within the ice in winter (through different trophic cascading effects). Timing of ice formation is critical in at least two ways: (i) an early ice formation could result in incorporation of more phytoplankton from fall blooms into the ice, (ii) more total light available for ice algal growth before mid-winter (Fig. 6a; see ref. 49). Thus, ice forming earlier would have higher concentrations of ice algae than later-forming ice, resulting in higher krill growth and survival rates^48^,^50^ (Fig. 6b). In turn, krill and/or non-euphausiid macrozooplankton and micronekton feeding under winter sea ice^26^,^28^,^29^ may supply the under-ice ecosystem up through to mesopelagic areas by transferring the energy to the pelagic food web (see schematic in Fig. 6c)^25^,^30^,^31^,^32^,^33^,^34^.

Female SESs demonstrate a more than 40% increase in their hunting times when foraging in years of earlier sea ice advance. This was associated with increased dive duration but slightly fewer dives per day (Supplementary information, figure S3). This result is in contrast to a recent study which found that earlier sea ice advance in the western Ross Sea region had a negative influence on the number of breeding seals from Macquarie Island, with a lag of 3 years^14^. They suggested earlier sea ice advance would prevent seals from accessing profitable prey patch areas close to the continental shelf or within the pack ice. These contrasting results in two different regions of Antarctica not only highlight the difficulty associated with simply extrapolating results from one region to another, but also underline the complex linkages between seal foraging performance and sea ice characteristics. Earlier advance of sea ice may have either a positive or negative influence on foraging depending on the current state of the environment. The increasing duration of the ice season has been particularly marked in the western Ross Sea sector over the past three decades^3^, to the point where the benefit of having a more developed ecosystem readily available earlier in the season could have been negated by the increasing constraints for air-breathing predators associated with higher concentrations of sea ice cover. In contrast, in the East Antarctic sector studied here, the inter-annual sea ice duration anomalies are subtler and generally less pronounced^4^, and an earlier start of the ice season is associated with longer hunting times and appears to benefit female SESs through increased foraging success. This situation could change, however, if season length were to considerably increase. While we found little evidence linking female foraging activity and sea ice concentration anomalies when sea ice seasonality was removed, we did find that female foraging activity increased in more concentrated sea ice, consistent with previous results^25^.

The linkages between sea ice and animal foraging activity are complex and dependent upon the regional setting and the spatio-temporal variability of the sea ice cover^13^,^25^,^51^. Earlier sea ice advance or increased sea ice concentration might be profitable only if SESs are able to access/locate profitable prey patches within sea ice. The Indian Ocean sector (20–90°E) is a region where many open ocean low concentration features occur in the ice pack associated with mesoscale eddies^52^. Also, the western Pacific Ocean sector (90°–160°E) is the least sea ice covered sector^53^ with generally divergent ice pack motion, dominated by leads and thin ice with a relatively large number of coastal polynyas^45^. Thus, this regional variability in sea ice across East Antarctica might allow predators to forage within sea ice covered areas. By contrast, high sea ice coverage and persistence such as in the Western Ross sea sector might impede access to the rich under-ice ecosystems within pack ice or in polynya areas^14^.

In contrast to females, male hunting times increased in years with lowest sea ice concentration, and the timing of sea ice advance had a weak effect on their foraging activity. In previous studies, we showed that males remain deeper in the sea ice zone and are able to forage on the Antarctic shelf and slope front region probably due to the presence of recurrent and persistent coastal polynyas and leads^20^,^25^. Antarctic coastal polynyas, often harbouring the highest phytoplankton biomass on the relatively productive continental shelf^54^, are sites of concentrated biological activity with rich ecosystems. As a result they support large populations of mammals that can breathe and feed throughout the ice season^55^. More work is necessary to investigate the nature and drivers of inter-annual variability/change in key coastal polynyas, and their relationship with wind strength and direction^56^, fast ice distribution^57^ and sea ice seasonality. One possible caveat is that satellite passive microwave retrieval of sea ice concentration in polynyas can be inaccurate due to the presence of extensive thin ice and coastal contamination^58^. This could compromise the accurate computation of the day of sea ice advance in polynya regions - to possibly explain why the timing of sea ice advance has an apparent significant but weak and counterintuitive effect on male foraging behaviour.

Both wind-driven dynamics and thermodynamic processes have played an important role in determining the regional complexity and variability of sea ice changes since 1979^39^. The strength of near-surface meridional winds increased female hunting times through earlier sea ice advance and increased sea ice concentrations outside polynyas and the biotic processes described above. No clear relationship was observed for males probably due to the complex influence of near-surface meridional wind anomalies on polynyas or open water areas close to the coast. Perhaps, once males are positioned in polynyas, wind-driven sea ice production and polynya size changes may not affect the prey availability or male foraging activity during the remainder of the winter season. These results compliment several studies emphasizing the complexity of wind-driven sea ice changes and its contrasting effects on Antarctic ecosystems. For example, winds (depending on strength and direction) can greatly affect higher-predator sea ice habitat by inducing: (i) ice convergence and compaction events^5^,^37^ leading to thicker ice and greater constraints for air breathing predators such as seals and whales^27^,^59^; (ii) loss of ice in other sectors, and loss of krill with negative effects on for example the krill-feeding crabeater seal (*Lobodon carcinophagus*)^9^; and (iii) spatio-temporal variability in fast ice distribution^60^, with contrasting effects e.g., on emperor penguins i.e., positive associated with larger polynyas or lower fast ice extent but also negative resulting from changes in fast ice persistence for breeding^10^.

We observed in the present study that both local anomalies of sea ice concentration and advance are (at least partly) controlled by local near-surface winds. Reference 61 has predicted a slight weakening of coastal surface winds during the 21^st^ century, becoming less katabatic in nature which may effect the “sea icescape”, prey availability and access for air breathing predators through the persistence and timing of polynya opening, sea ice expansion and thinning. Although highly speculative, it is interesting to put these predicted changes in the context of the results presented in this study. Weakening of katabatic winds probably inducing later sea ice advance and decreased sea ice concentration might affect predator foraging success since Antarctic ecosystems are not only adapted to sea ice presence but also to its seasonal rhythms and properties^5^. However, it is important to consider that seals may have the behavioural flexibility or adaptive capacity to cope with long term changes^62^.

Our study describes for the first time the significant combined effects of the inter-annual variability of near-surface winds as they affect sea ice coverage on the foraging activity of a predator (based upon an 11-year time series). It has also proposed mechanisms by which climate forcing affects both abiotic and biotic components of the Antarctic marine ecosystem. Understanding responses to environmental change is particularly important in the case of predators, which play crucial roles in regulating ecosystems^63^. The spatial heterogeneity of sea ice changes in East Antarctica^4^ makes this region unique for our understanding of ecological processes taking place between top predators and sea ice changes. We have proposed mechanisms by which sea ice changes might have direct effects on top predators through trophic cascading processes. Finally, this work highlights the lack of information on ecological processes taking place in the under-ice ecosystems up to mesopelagic areas, and in winter in particular.

## Methods

### Animal handling, deployment, data collected and filtering

We use location and dive depth data from 43 post-moulting SESs (21 females and 22 males) that were instrumented with CTD-SRDLs (Sea Mammal Research Unit, University of St Andrews) between December and February in 2004, 2008–2009 and 2011–2014 on the Kerguelen Islands (49°20′S, 70°20′E) (Supplementary information, Table S1). These animals were chosen from a larger dataset because they visited the area south of 55°S (the spatial domain for the study), which is equivalent to the maximum latitude of annual sea ice extent (in September). Unusual behaviour was observed in five animals (two females and three males) that returned to the colony before heading back to sea again. For these individuals, the section of the tracks where the animals travelled south within the sea ice region (one female and two males) after their return to the colony were removed from the analysis. Details of the instrumentation, seal handling and data processing for dives and filtering ARGOS positions are provided in ref. 20. All animals in this study were handled in accordance with the French Polar Institute (Institut Paul Emile Victor, IPEV) ethical and Polar Environment Committees guidelines associated with the research project IPEV # 109 (PI H. Weimerskirch). The experimental bio-logging protocol was approved by the IPEV ethical and Polar Environment Committees.

### Foraging activity

Foraging activity of each seal was analysed at the dive scale using the methodology developed by ref. 47, which estimates the time spent hunting during a dive. For each dive, the time spent in segments with a vertical velocity lower or equal to 0.4 m.s^−1^ was calculated. This time was termed hunting time per dive and was used as a proxy for foraging activity.

### Sea ice concentration anomalies

SSM/IS daily sea ice concentration (resolution 25 km) provided a continuous time-series for the years of the study. The mean seasonal cycle of sea ice concentration was produced by averaging daily maps corresponding to the same day of year, over the 11 years of the study. Once the seasonal cycle was computed from this time series, we then removed this signal from the time series of sea ice concentration, to create an anomaly from the local seasonal cycle.

In order to test relationships between daily sea ice concentration anomalies and seal hunting times, we grouped all seal hunting times corresponding to the location of anomalously negative and positive sea ice concentrations (defined as a sea ice concentration anomaly lower or greater than one standard deviation of sea ice concentration anomaly respectively). For this calculation, we only considered seals inside the sea ice region (as defined by their distance to the sea ice edge, i.e. 15% ice concentration isoline) and from March onward, as previously defined. We then compared the two distributions of hunting times using a permutation test (bootstrap analysis)^64^. We repeated the experiment of grouping seals hunting time 10,000 times, but randomly selected seals in our dataset, *i.e.* independent of collocated sea ice concentration anomalies. We then compared the distribution of the 10,000 differences in hunting time from the 10,000 random pairs of groups, to the difference of hunting time from the two groups based on sea ice concentration anomalies. To take into account individual variability, we computed two more tests, presented in the Supplementary information. First a permutation test by individual was applied (i.e. instead of grouping hunting times 10,000 times by randomly selected observations in our dataset, all seals combined), we grouped hunting times 10,000 times by randomly selected observations for each seal one by one. Then we compared the distribution of the 10,000 differences in hunting time from the 10,000 random pairs of groups selected from one given seal, to the observed difference of hunting time from the two groups based on sea ice concentration anomalies from the same seal. This procedure was repeated for each individual. Second, a permutation test by bootstrapping hunting times at the individual level 100 times was applied: we first sampled with replaced animals among the pool of observed SESs and included all observations from these randomly sampled individuals. For each bootstrap sample, n random individuals from our dataset were selected leading to sampling for example several times the same individual and removing some others. We then compared the distribution of the 100 differences in hunting time from the 100 random pairs of groups for sea ice concentrations anomalies.

Finally, SSMI/S monthly sea ice concentration (resolution 25 km) were used to compute monthly sea ice concentration anomalies to perform the correlation with monthly wind anomalies (see section *Surface wind anomalies*).

### Sea ice advance anomalies

The day at which sea ice advances in the season (hereafter referred to as sea ice advance) was derived following ref. 4 using the NASA Bootstrap SMMR-SSM/I NASA Team combined dataset of daily sea ice concentration^65^ (http://nsidc.org/data/nsidc-0051.html) with a resolution of 25 km. Following ref. 41, the day of ice advance is taken to be the time at which sea ice concentration in a given pixel first exceeds 15% (proxy for the ice edge) for at least 5 consecutive days, for a given sea ice year (twelve months from mid-February). We computed the sea ice advance anomalies (from the local seasonal cycle) by removing the local climatological seasonal cycle computed over the 11 years of the study. We then collocated the sea ice advance anomalies at each seal position and time.

The goal of computing sea ice advance anomalies was to determine any possible influence of relatively early or late sea ice advance on seal foraging behaviour. The period during which seal hunting behaviour is likely to be affected by an earlier or later sea ice advance would be around the time of year at which sea ice usually advances. We therefore only selected seals’ positions during the seasonal advance of sea ice from March to June^66^ that occurred within a 30-day window around the day of sea ice advance for a given year and at a given pixel. From this sub-sample, we compared the hunting time distribution for the two groups of seals i.e., those associated with later advance (i.e. positive anomalies), and those with earlier advance (i.e. negative anomalies). Similar to the sea ice concentration anomaly procedure, we estimated the significance of the difference in hunting time for the two groups using a permutation test and we took into account individual variability with the two analyses described above.

### Surface wind anomalies

Surface zonal and meridional winds were extracted from monthly ERA-Interim 10 m atmospheric reanalysis (http://apps.ecmwf.int/datasets/) with a spatial resolution of approximately 80 km. We computed meridional wind anomalies (from the local seasonal cycle) by removing the local climatological seasonal cycle computed over the 11 years of the study. The relationship between monthly meridional wind anomalies and monthly sea ice concentration anomalies was likely to be non-linear, so the correlation for each longitude/latitude pixel over the 11 year time period was performed using a Spearman correlation. For both variables, the periodic inter-annual variability was taken into account and the first trend in the anomalies was removed prior to correlation. The relationship between monthly meridional wind anomalies and sea ice advance anomalies was processed in three steps: (i) for each 5° longitude bin, monthly ERA-Interim 10 m wind anomalies were averaged within the minimum and maximum latitude band of average day of advance from 2004 to 2014; then (ii) the resulting averaged winds per month for each 5° longitude were averaged from March to June to obtain one-yearly data per bin of longitude; and finally (iii) a Spearman correlation between sea ice advance anomalies and averaged wind anomalies for each 5° bin of longitude was computed.

### Statistical modelling

A Gaussian additive mixed effects model (GAMM) was first fitted to examine the statistical relationships between seal foraging activity (expressed by the hunting time per dive) and the 10 m wind anomaly meridional component data. Because the estimated relationship with a GAMM was linear, we then fitted a linear mixed effects model (LMM). Monthly ERA-Interim 10 m wind anomalies were collocated at each seal position and time. A subset of the data was extracted to only focus on parts of the tracks influenced by sea ice; for this, only positions inside the sea ice and from March (when the seasonal signal of sea ice concentration starts to increase^66^) to the end of the post-moult trip were used for subsequent analysis. For each individual within a given month, hunting times per dive and monthly 10 m wind collocated at the seal dive position were averaged monthly. Models were computed with the R packages *mgcv* and *nlme* (from R Development Core Team, function *gamm* and *lme*) using restricted maximum likelihood. Outliers in the variables were checked. Sex was included in the model as an interaction factor variable. We first determined the optimal structure by assessing if individual seals as a random intercept term and if monthly 10 m wind (collocated at the seal dive position and averaged monthly) as a random slope term contributed to the model fit. The final model was then fitted using restricted maximum likelihood (REML). Model validation was checked by plotting Pearson residuals against fitted values, and against the explanatory variable, to verify homogeneity and normality of residuals^67^ (Supplementary information, figures S4 and S5). Finally, a marginal R-squared (i.e. variance explained by fixed factors only) and a conditional R-Squared (i.e. variance explained by both fixed and random factors) were calculated using the R package *MuMIn* (from R Development Core Team, function *r.squaredGLMM*).

## Additional Information

**How to cite this article**: Labrousse, S. *et al*. Variability in sea ice cover and climate elicit sex specific responses in an Antarctic predator. *Sci. Rep.* **7**, 43236; doi: 10.1038/srep43236 (2017).

**Publisher's note:** Springer Nature remains neutral with regard to jurisdictional claims in published maps and institutional affiliations.

## Supplementary Material

Supplementary Information

## Figures and Tables

**Table 1:** Summary of the difference in hunting times between negative and positive sea ice concentration anomalies and earlier and later sea ice advance for males and females.

	Negative sea ice concentration anomalies (Females = 15; Males = 18)	Positive sea ice concentration anomalies (Females = 14; Males = 17)	Total_males_	Total_females_
Males	4.6 min longer/dive (~47% of the median hunting time)	n.a	21	
Males with (ID-V)	4.9 min longer/dive (~50% of the median hunting time)	n.a		
Females	n.a	3.9 min longer/dive (~24% of the median hunting time)		17
Females with (ID-V)	n.a	4.6 min longer/dive (~28% of the median hunting time)		
	**Earlier sea ice advance (Females = 14; Males = 17)**	**Later sea ice advance (Females = 14; Males = 16)**	**Total**_**males**_	**Total**_**females**_
Males	1.9 min longer/dive (~20% of the median hunting time)	n.a	19	
Males with (ID-V)	0.6 min longer/dive (~6.6% of the median hunting time)	n.a		
Females	5.3 min longer/dive (~41% of the median hunting time)	n.a		14
Females with (ID-V)	3.5 min longer/dive (~30% of the median hunting time)	n.a		

Values were indicated for all individuals or for randomly selected individuals among the pool of observed SESs to take into account individual variability (ID-V). The number of individuals in each group and the total was also indicated.
