# Supplementary material for: Variability in sea ice cover and climate elicit sex specific responses in an Antarctic predator
Source: Sci Rep. 2017 Feb 24;7:43236. doi: 10.1038/srep43236 (PMC5324094; doi:10.1038/srep43236)
Supplement: Supplementary Information [file srep43236-s1.pdf]

Variability in sea ice cover and climate elicit sex specific responses in an Antarctic predator

Labrousse, Sara[1, 2*]; Sallée, Jean-Baptiste[1, 3]; Fraser, Alexander D.[4, 5]; Massom, Rob A.[5, 6]; Reid, Phillip[7]; Hobbs, William[5, 8]; Guinet, Christophe[9]; Harcourt, Robert[10]; McMahon, Clive[2, 10, 11]; Authier, Matthieu[12]; Bailleul, Frédéric[13]; Hindell, Mark A.[2, 5]; Charrassin, Jean-Benoit[1].

*Correspondence to sara.labrousse@gmail.com

*(1) Sorbonne Universités, UPMC Univ., Paris 06, UMR 7159 CNRS-IRD-MNHN, LOCEAN-IPSL, 75005 Paris, France*

*(2) Marine Predator Unit, Institute for Marine and Antarctic Studies, University of Tasmania, Private Bag 129, Hobart, Tasmania 7001, Australia*

*(3) British Antarctic Survey, High Cross, Cambridge, CB3 0ET, UK*

*(4) Institute of Low Temperature Science, Hokkaido University, N19 W8, Kita-ku, Sapporo, Japan 060-0819*

*(5) Antarctic Climate & Ecosystems Cooperative Research Centre, University of Tasmania, Private Bag 80, Hobart, Tasmania 7001, Australia*

*(6) Australian Antarctic Division, Channel Highway, Kingston, Tasmania 7050, Australia*

*(7) Australian Bureau of Meteorology, Centre for Australian Weather and Climate Research, Hobart, Tasmania 7001, Australia*

*(8) Centre of Excellence for Climate System Science, Australian Research Council, Sydney, New South Wales 2052, Australia*

*(9) Centre d'Etudes Biologiques de Chizé (CEBC), UMR 7372 Université de la Rochelle-CNRS, 79360 Villiers en Bois, France*

*(10) Department of Biological Sciences, Macquarie University, Sydney, New South Wales 2109, Australia*

*(11) Sydney Institute of Marine Science, 19 Chowder Bay Road, Mosman, New South Wales 2088, Australia*

*(12) Observatoire PELAGIS, UMS 3462 CNRS-ULR, 17000 La Rochelle, France*

*(13) South Australian Research & Development Institute (SARDI), 2 Hamra Avenue, West Beach, South Australia 5024, Australia*

**Supplementary information**

**Table S1 | General information of the 43 post-moulting SESs (22 males and 21 females).** It includes sex, dive start and end date, date of return when the tag did not stopped, number of Argos position transmitted daily, animal weight and snout-to-tail length upon deployment, total number of dives, mean number of dives per day and mean distance travelled per day between the first and last locations of each day. Additional information on behaviour towards sea ice is also included such as their maximal distance from the sea ice edge and their hunting time per dive. Negative distances refer to distances into the pack from the ice edge, and positive distances refer to distances north of the ice edge. Mean are expressed ± SD. Finally, individuals not included in all analysis are detailed in the analysis column.

| ID | Sex | Dive start date | Dive end date | Date of return to the colony (if tag did not stopped) | Number of position transmitted daily | Weight (kg) | Length (cm) | Total dives | Daily number of dives | Distance travelled per day (km) | Maximal distance from the sea ice edge (km) | Hunting time per dive within sea ice from March (min) | Analysis |
|---|---|---|---|---|---|---|---|---|---|---|---|---|---|
| 2004_1 | M | 04/03/2004 | 29/03/2004 | | 8 ± 4 | 368 | 250 | 553 | 25 ± 12 | 75 ± 49 | -62 | 5,4 ± 3,9 | × |
| 2004_2 | M | 27/02/2004 | 09/07/2004 | | 18 ± 7 | 385,5 | 267 | 6133 | 46 ± 20 | 34 ± 33 | -192 | 11,3 ± 7,3 | × |
| 2004_3 | F | 01/03/2004 | 19/07/2004 | | 14 ± 9 | 297,5 | 233 | 5363 | 38 ± 20 | 34 ± 29 | -345 | 14,6 ± 11,3 | × |
| 2004_5 | M | 25/02/2004 | 06/08/2004 | | 17 ± 6 | 469,5 | 282 | 7209 | 46 ± 18 | 22 ± 31 | -341 | 14 ± 10 | × |
| 2004_6 | F | 22/02/2004 | 07/08/2004 | | 12 ± 6 | 347 | 240 | 4248 | 27 ± 12 | 28 ± 26 | -165 | 22 ± 10,2 | × |
| 2004_7 | F | 29/02/2004 | 02/08/2004 | | 15 ± 9 | 295,5 | 238 | 6021 | 40 ± 19 | 42 ± 28 | -110 | 8,5 ± 4 | × |
| 2004_8 | M | 27/02/2004 | 08/08/2004 | 6 then South | 17 ± 9 | 274 | 235 | 7530 | 50 ± 25 | 40 ± 34 | -610 | 5,4 ± 4,7 | × |
| 2004_10 | F | 29/02/2004 | 08/08/2004 | | 16 ± 10 | 363,5 | 258 | 7503 | 46 ± 24 | 31 ± 29 | -367 | 13 ± 10 | × |
| 2008_1 | M | 01/01/2008 | 08/09/2008 | | 9 ± 5 | 266 | 230 | 8815 | 39 ± 30 | 33 ± 26 | -161 | 10 ± 7,5 | × |
| 2008_2 | F | 24/12/2007 | 27/05/2008 | | 14 ± 7 | 169 | 200 | 6031 | 39 ± 16 | 44 ± 30 | -8 | - | Only used in sea ice advance analysis |
| 2008_6 | F | 24/01/2008 | 16/08/2008 | | 11 ± 4 | 290 | 242 | 6200 | 31 ± 10 | 42 ± 26 | -3 | 11,3 ± 6,2 | × |
| 2008_7 | F | 27/01/2008 | 11/07/2008 | | 15 ± 7 | 377 | 267 | 5253 | 32 ± 13 | 44 ± 32 | -244 | 17 ± 9,8 | × |
| 2009_16 | M | 01/01/2009 | 03/06/2009 | 6 | 17 ± 7 | 258 | 249 | 5887 | 40 ± 18 | 34 ± 28 | -155 | 9,4 ± 7 | × |
| 2011_4 | M | 31/01/2011 | 16/05/2011 | | 26 ± 7 | 800 | 330 | 4438 | 42 ± 11 | 33 ± 39 | -316 | 13,5 ± 7,1 | × |
| 2011_6 | F | 19/02/2011 | 16/05/2011 | | 31 ± 9 | 284,6 | 233 | 4230 | 50 ± 11 | 32 ± 31 | -4 | 10,6 ± 5,8 | Absent in sea ice advance analysis |
| 2011_7 | M | 26/01/2011 | 15/04/2011 | | 34 ± 10 | 452,5 | 280 | 4749 | 60 ± 19 | 36 ± 39 | -302 | 9,3 ± 6,5 | × |
| 2011_9 | M | 27/01/2011 | 16/05/2011 | | 18 ± 6 | 628,5 | 326 | 3487 | 32 ± 12 | 29 ± 37 | -409 | 14,6 ± 9 | × |
| 2011_10 | F | 24/02/2011 | 16/05/2011 | | 20 ± 9 | 330 | 250 | 3041 | 37 ± 11 | 35 ± 28 | -37 | 14,5 ± 8 | × |
| 2012_1 | M | 23/01/2012 | 14/09/2012 | | 18 ± 6 | 523 | 291 | 9799 | 43 ± 18 | 31 ± 28 | -434 | 10,6 ± 11,1 | × |
| 2012_3 | M | 23/01/2012 | 26/04/2012 | | 24 ± 6 | 454 | 277 | 4297 | 45 ± 11 | 36 ± 38 | -286 | 13,2 ± 6,2 | × |

| | | | | | | | | | | | | | |
|---|---|---|---|---|---|---|---|---|---|---|---|---|---|
| 2012_2 | F | 07/02/2012 | 28/09/2012 | 9 | 20 ± 9 | 303 | 233 | 7178 | 31 ± 12 | 28 ± 21 | -58 | 17 ± 9,1 | × |
| 2013_1 | F | 27/02/2013 | 19/10/2013 | 10 | 18 ± 6 | 340 | 262 | 8079 | 34 ± 9 | 43 ± 30 | -130 | 17,5 ± 10,4 | × |
| 2013_2 | M | 08/03/2013 | 02/11/2013 | 11 | 17 ± 10 | 1100 | 370 | 8321 | 39 ± 17 | 33 ± 41 | -482 | 17,4 ± 10,5 | × |
| 2013_3 | M | 10/02/2013 | 17/03/2013 | | 22 ± 9 | 468 | 280 | 1513 | 46 ± 9 | 67 ± 41 | -140 | 7,2 ± 5,7 | × |
| 2013_4 | M | 03/03/2013 | 09/09/2013 | 9 | 18 ± 7 | 850 | 333 | 6064 | 35 ± 12 | 36 ± 36 | -699 | 18,1 ± 11,5 | × |
| 2013_5 | F | 24/02/2013 | 17/12/2013 | 9 then South | 22 ± 8 | 336 | 254 | 11732 | 43 ± 16 | 29 ± 27 | -745 | 16 ± 11,9 | × |
| 2013_7 | F | 17/02/2013 | 13/10/2013 | | 19 ± 7 | 410 | 248 | 9204 | 42 ± 14 | 43 ± 36 | -256 | 15,1 ± 10,7 | × |
| 2013_9 | M | 11/02/2013 | 14/03/2013 | | 24 ± 6 | 470 | 300 | 1517 | 47 ± 15 | 63 ± 45 | -157 | 9,1 ± 5,8 | × |
| 2013_11 | M | 11/02/2013 | 08/10/2013 | | 23 ± 7 | 556 | 256 | 10151 | 44 ± 13 | 22 ± 32 | -962 | 12 ± 8,1 | × |
| 2013_12 | M | 17/02/2013 | 07/10/2013 | 10 | 19 ± 7 | 1150 | 375 | 7728 | 36 ± 12 | 31 ± 21 | -164 | 23,3 ± 10 | × |
| 2013_13 | M | 10/02/2013 | 20/04/2013 | | 23 ± 6 | 600 | 321 | 3501 | 50 ± 17 | 50 ± 37 | -221 | 6,8 ± 6 | × |
| 2013_14 | M | 17/03/2013 | 24/11/2013 | 11 | 20 ± 8 | 300 | 270 | 10074 | 42 ± 16 | 19 ± 32 | -743 | 15 ± 11,2 | × |
| 2013_15 | F | 10/02/2013 | 29/09/2013 | 10 | 20 ± 7 | 366 | 248 | 8335 | 38 ± 9 | 47 ± 26 | -121 | 17,8 ± 10,3 | × |
| 2013_18 | F | 07/02/2013 | 03/08/2013 | | 23 ± 9 | 346 | 255 | 6723 | 41 ± 15 | 34 ± 30 | -192 | 21,6 ± 8,5 | × |
| 2014_2 | F | 25/01/2014 | 30/03/2014 | | 24 ± 10 | 304 | 255 | 2793 | 48 ± 15 | 56 ± 31 | -34 | 8,3 ± 6,3 | × |
| 2014_3 | F | 25/01/2014 | 04/10/2014 | 10 | 16 ± 6 | 293 | 244 | 7038 | 29 ± 8 | 28 ± 21 | -64 | 28 ± 10,1 | × |
| 2014_4 | F | 30/01/2014 | 12/03/2014 | | 22 ± 9 | 265 | 236 | 1840 | 45 ± 13 | 57 ± 32 | 31 | - | Only used in sea ice advance analysis |
| 2014_6 | F | 28/01/2014 | 30/09/2014 | 9 | 19 ± 6 | 266 | 243 | 8241 | 36 ± 10 | 32 ± 23 | -128 | 22,7 ± 9,2 | × |
| 2014_7 | M | 26/12/2013 | 23/10/2014 | 7 then South | 19 ± 9 | 405 | 277 | 11722 | 46 ± 21 | 32 ± 32 | -857 | 9,1 ± 8,3 | × |
| 2014_8 | F | 30/01/2014 | 21/09/2014 | | 17 ± 6 | 270 | 247 | 7249 | 34 ± 10 | 28 ± 25 | -203 | 21,2 ± 10,1 | × |
| 2014_9 | M | 29/12/2013 | 11/09/2014 | | 12 ± 6 | 700 | 322 | 4233 | 22 ± 10 | 35 ± 32 | -195 | 23,5 ± 11,1 | × |
| 2014_10 | M | 27/12/2013 | 27/09/2014 | 6 then North | 14 ± 8 | 700 | 306 | 7876 | 35 ± 14 | 27 ± 36 | -241 | 14,5 ± 8,7 | × |
| 2014_11 | F | 29/01/2014 | 17/09/2014 | | 24 ± 13 | 295 | 249 | 8346 | 38 ± 19 | 28 ± 26 | -148 | 14,7 ± 9,2 | × |
| Mean ± SD or sum | – | – | – | | 18 ± 9 | – | – | 273542 | 39 ± 17 | 34 ± 31 | | 14 ± 10 | – |
| Mean ± SD or sum males | – | – | – | | – | 554 ± 248 | 292 ± 40 | 135534 | 41 ± 19 | 32 ± 34 | -370 ± 254 (min males = -962) | 13 ± 10 | _ |
| Mean ± SD or sum females | – | – | – | | – | 312 ± 51 | 245 ± 14 | 138008 | 38 ± 15 | 36 ± 28 | -159 ± 174 (min females = -745) | 17 ± 11 | _ |

**Individual variability in seal foraging activity response to inter-annual sea ice cover anomaly**

**Males**

When taking into account the individual variability in the analysis, only 14/21 males were foraging in both negative and positive SIC anomalies and among these individuals the same relation (i.e. hunting times were longer in years with lower sea ice concentration) was observed on 9/14 males and was significant for 9/14 males (Figure S1a). When bootstrapping at the individual level for males, the same relation was observed with hunting times 4.9 min/dive longer in negative sea ice concentration anomalies (50% of the median hunting time; Figure S2a), confirming the significance of the relation despite individual variability.

Regarding the effect of the timing of sea ice advance on male foraging activity when taking into account the individual variability in the analysis, only 14/19 males were foraging in both groups of earlier and later sea ice advance and among these individuals higher hunting times in earlier sea ice advance was only observed for 5/14 males and significant for only 2/14 males. This confirmed the weak effect of the timing of sea ice advance on males in this study (Figure S1c). Moreover, when bootstrapping at the individual level for males, the difference between hunting times was very low (i.e. 0.6 min/dive longer, 7% of the median hunting time; Figure S2c), confirming a second time the weakness of this relation for males and the important individual variability.

**Females**

When taking into account the individual variability in the analysis, only 12/17 females were foraging in both negative and positive SIC anomalies and among these individuals the same relation (i.e. hunting times were longer in years with higher sea ice concentration) was observed on 8/12 females and was significant for 5/12 females (Figure S1b). When bootstrapping at the individual level for females, the same relation was observed with hunting times 4.6 min/dive longer (28% of the median hunting time; Figure S2b), confirming the significance of the relation despite individual variability.

Regarding the effect of the timing of sea ice advance on female foraging activity when taking into account the individual variability in the analysis, 14/14 females were foraging in both groups of earlier and later sea ice advance and among these individuals higher hunting times in earlier sea ice advance was observed for 9/14 females and significant for 7/14 females. This confirmed the important effect of earlier sea ice advance on the majority of individuals (Figure S1d). However, when bootstrapping at the individual level for females, the same relation was observed but the difference in hunting times was lower (i.e. 3.5 min/dive longer, 30% of the median hunting time; Figure S2d), suggesting that individual variability was driving in part the relation.

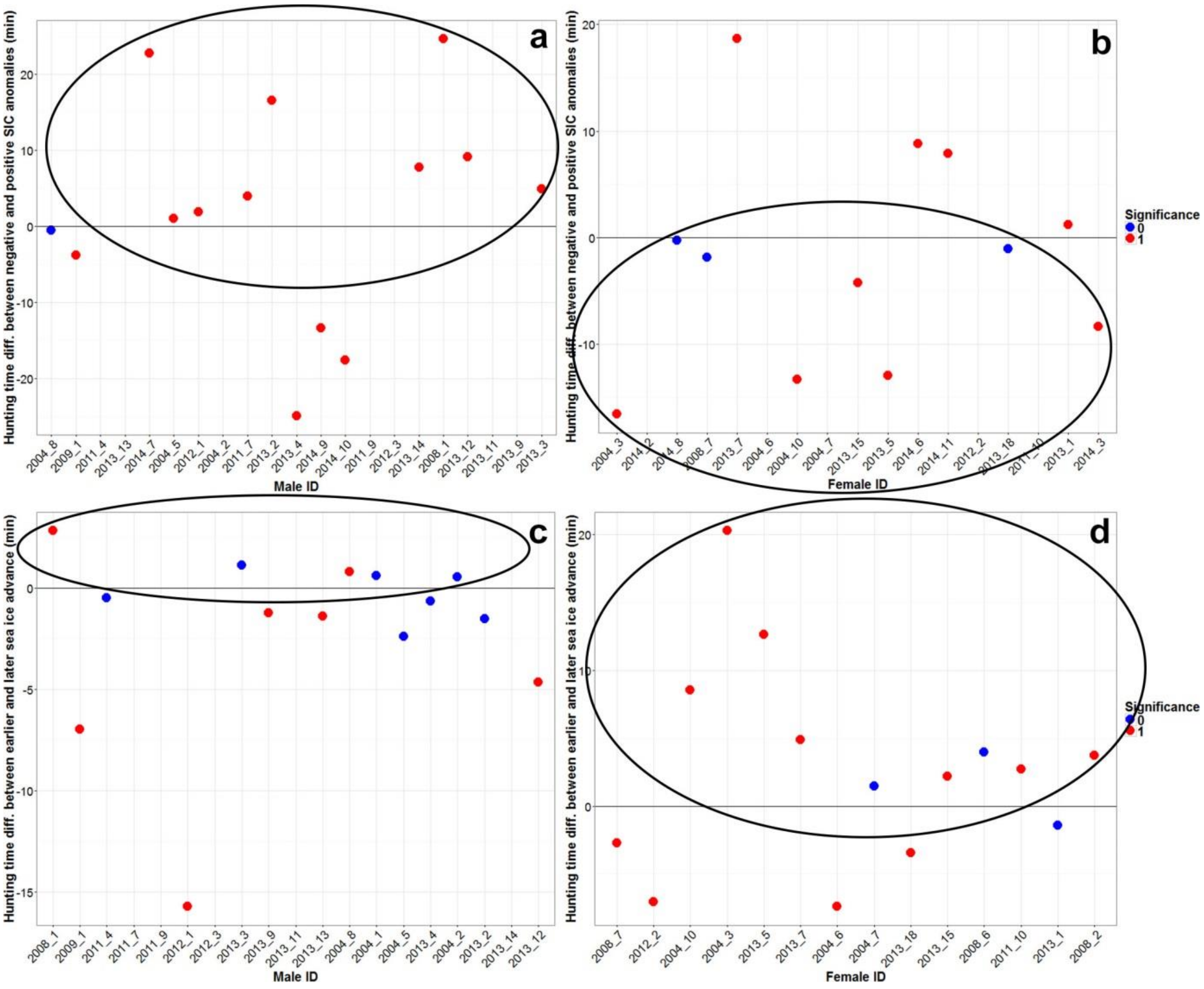


**Figure S1 | Individual variability of the influence of sea ice changes on male and female foraging activity from 2004 to 2014.** Observed differences between negative and positive sea ice concentration anomalies per individual are represented for the (**a**) 21 males and (**b**) 17 females. Observed differences between earlier and later sea ice advance per individual are represented for the (**c**) 19 males and the (**d**) 14 females. Significance of the relation was obtained from the comparison of the distribution of the 10,000 differences in hunting time from the 10,000 random pairs of groups, to the observed difference of hunting time from the two groups based on sea ice concentration anomalies for each individual. Significant relations are represented by a red dot (i.e. $p\text{-value} < 0.05$) in contrast to blue dot representing non-significant relations (i.e. $p\text{-value} \geq 0.05$). Absence of dots for some individuals means they used only one type of sea ice groups along their total trip. Ellipses represent where most seals should be based on the relation computed on all individuals.

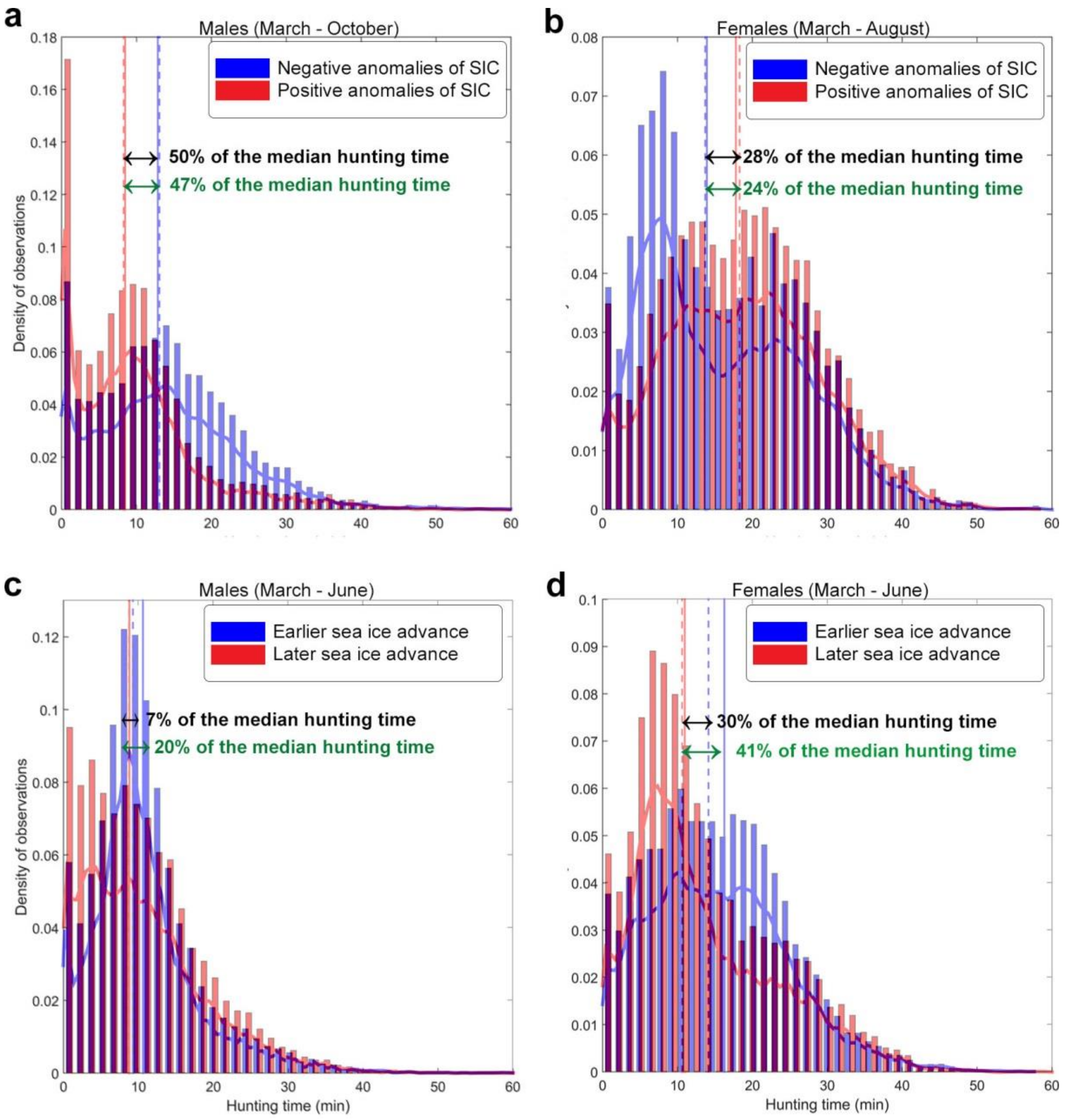


**Figure 2 | Influence of sea ice changes on male and female foraging activity from 2004 to 2014 taking into account individual variability.** Normalized histograms of the sum of observations in each bin of hunting time (*i.e.* a proxy of seal foraging activity expressed in minutes) are represented for negative or positive sea ice concentration anomalies (see Methods) for 100 bootstrap samples. For each bootstrap sample, sample composition was randomized at the individual level to assess whether the observed population-level difference could be due to between-individual variability. We randomly selected with replacement **(a)** 21 males among the 21 males and **(b)** 17 females among 17 females. The same histograms are presented for earlier and later advance of sea ice for 100 bootstrap samples where we randomly selected with replacement **(c)** 19 males among the 19 males and **(d)** 14 females among the 14 females. For each group of anomalies, the probability density function was superimposed and the dashed lines represent the bootstrapped median hunting time for each group of anomalies for males and females. Continuous lines represent the observed median hunting times when selecting all individuals, not taking in account individual variability (as presented in Fig. 3 of the main text). The percentages of difference in hunting times between two groups are written in black for the bootstrap samples and in green for the observed sample: except for the effect of sea ice advance on males, the bootstrap and observed samples were similar, suggesting that the difference in hunting was not the sole result of individual. Please note that hunting times equal to 0 were removed for illustration purposes.

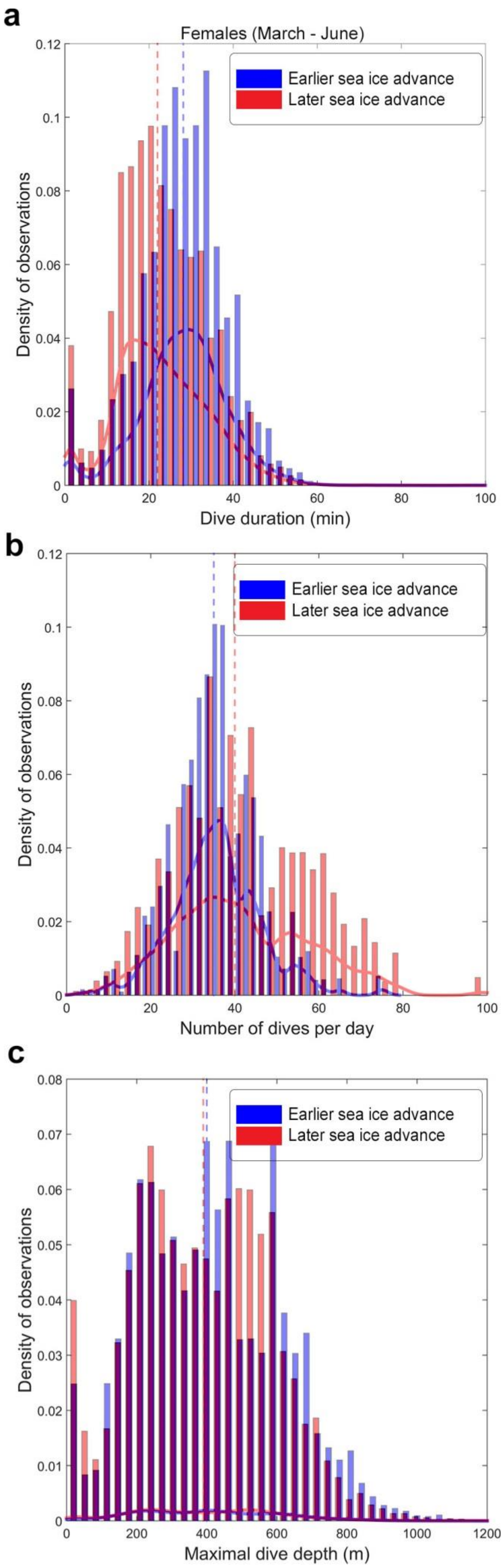
a
Females (March - June)
Earlier sea ice advance
Later sea ice advance
Density of observations
Dive duration (min)
b
Earlier sea ice advance
Later sea ice advance
Density of observations
Number of dives per day
c
Earlier sea ice advance
Later sea ice advance
Density of observations
Maximal dive depth (m)

**Figure S3 | Influence of the timing of sea ice advance on female diving behaviour from 2004 to 2014.** Normalized histograms of the sum of observations in each bin of (**a**) dive duration (expressed in minutes), (**b**) the number of dives per day and (**c**) the maximal dive depth (expressed in meters) are represented for earlier and later advance of sea ice. For each group of anomalies, the probability density function was superimposed and the dashed lines represent the median of each parameter for each group of anomalies for females.

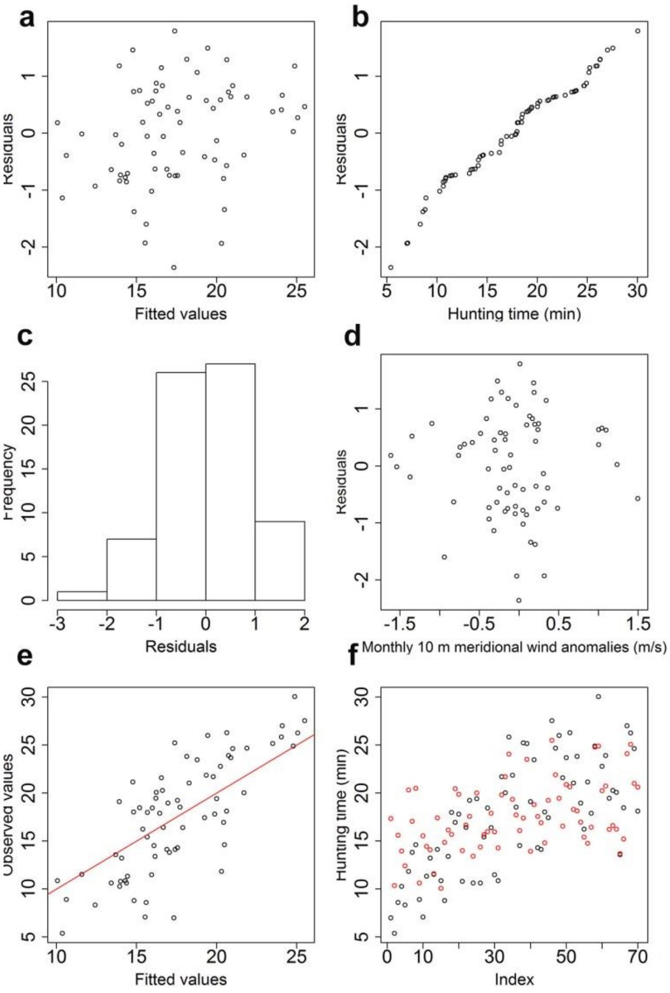


**Figure S4 | Diagnostic of the model examining the relationships between foraging activity and meridional near-surface wind anomalies for females at the monthly scale**. To verify the homogeneity and normality of residuals Pearson residuals were plotted against fitted values (**a**), against the explanatory variable (**d**), their distribution was drawn on a histogram (**c**) and on a normal QQ plot (**b**). Finally to examine the quality of the prediction, observed values were plotted against fitted values (**e**) and their distribution were superimposed (**f**) with black dots observed values and red dots fitted values.

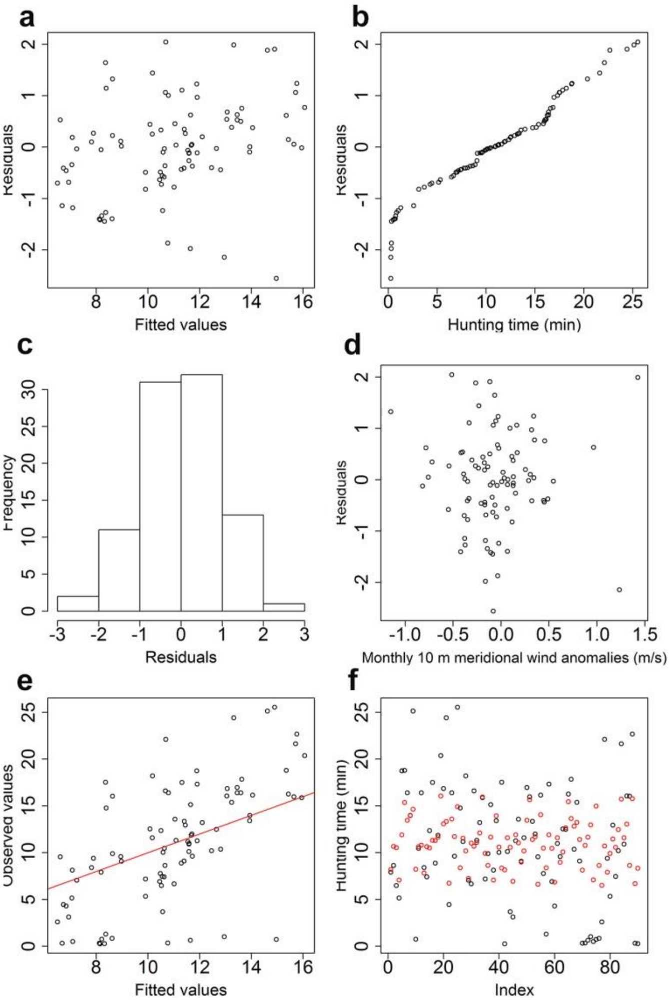


**Figure S5 | Diagnostic of the model examining the relationships between foraging activity and meridional near-surface wind anomalies for males at the monthly scale**. To verify the homogeneity and normality of residuals Pearson residuals were plotted against fitted values (**a**), against the explanatory variable (**d**), their distribution was drawn on a histogram (**c**) and on a normal QQ plot (**b**). Finally to examine the quality of the prediction, observed values were plotted against fitted values (**e**) and their distribution were superimposed (**f**) with black dots observed values and red dots fitted values.
